# Stepping into the shoes of older people: a scoping review of simulating ageing experiences for healthcare professional students

**DOI:** 10.1093/ageing/afad235

**Published:** 2023-12-28

**Authors:** Elaine E C Nelson, Andrew D Spence, Gerard J Gormley

**Affiliations:** Centre for Medical Education, Queen’s University Belfast, Belfast BT7 1NN, Northern Ireland; Centre for Medical Education, Queen’s University Belfast, Belfast BT7 1NN, Northern Ireland; Centre for Medical Education, Queen’s University Belfast, Belfast BT7 1NN, Northern Ireland

**Keywords:** simulation, healthcare students, older people, review

## Abstract

**Background:**

Ageing simulation suits and equipment give healthcare professional (HCP) students the opportunity to experience what it might feel like to be an older person with age-related disability or illness. Ageing simulation experiences, where students complete activities of daily living (ADL) tasks, aim to reproduce the challenges an older person may face.

**Objectives:**

We undertook a scoping literature review to establish, from the evidence base, what is known about simulating ageing experiences for HCP students and its impact on attitudes towards older patients.

**Methods:**

We applied Arksey and O’Malley’s scoping literature review framework to achieve relevant articles. Four databases (MEDLINE, Embase, Web of Science and Cumulative Index to Nursing and Allied Health Literature) were searched resulting in 114 citations. After screening and applying our exclusion criteria, 14 articles were selected for inclusion.

**Results:**

Fifty percent of studies were mixed-methods, 35% quasi-experimental, 7% quantitative and 7% qualitative. Two types of simulation experience were identified: (i) workshop based and (ii) ageing games. Simulated impairments included vision, hearing and mobility issues. Most common ADLs simulated were managing medications, finances and functional ability. The majority of studies reported a positive impact on knowledge, attitudes and empathy towards older people.

**Conclusions:**

Teaching in Care of Older People is important in HCP undergraduate curricula and should be a positive experience promoting successful ageing while raising awareness of ageism. Ageing suits have a positive impact on students’ attitudes and empathic skills towards older people. Future research should include interprofessional education with HCP students learning together throughout undergraduate training.

## Key Points

- Teaching in Care of Older People should be designed for all healthcare professional (HCP) undergraduate curricula.
- Educators must use their teaching skills to attract and inspire HCP students into Care of Older People.
- Ageing simulation provides an empathetic learning opportunity for students to reflect on challenges faced by older people.
- Future research should include interprofessional education between all HCP students.
- Interprofessional education is an opportunity to gain experience of collaborative Multidisciplinary Team working.

## Introduction

### Ageing and ageism

The average age of our society is rising. Although life expectancy is increasing, life quality is not rising at the same rate [1]. There are increasing pressures within the National Health Service to deliver health and social care to older people. To address this, there is a focus on ‘*Healthy Ageing*’, which is defined by the World Health Organisation (WHO) as ‘the process of developing and maintaining the functional ability that enables wellbeing in older age’ [2]. This consists of the older person’s intrinsic mental and physical capacities to maintain their basic needs and mobility, make decisions, maintain relationships and contribute to society. These intrinsic capacities interact with their environment at home and in the community. Having a supportive environment, which includes access to healthcare to maintain functional ability is key to healthy ageing. To provide support for the growing older population, the United Nations proposed this report: ‘Decade of Healthy Ageing’ (2021–2030) [3]. This includes the development of a ‘sustainable, appropriately trained, deployed and managed health workforce’ that is competent in ageing [3]. The WHO defines ageism as ‘stereotypes, prejudice and discrimination towards others or oneself based on age’ [4]. Ageism influences all aspects of older people’s health [5]. Wynam *et al*. showed age discrimination is common and established in healthcare professionals (HCPs) concluding that improved education and training of ‘key players’, i.e. hospital administrators, caregivers and HCPs is vital to reducing age bias [6].

We, as clinicians, medical leaders and educators, have a responsibility to HCP students to be a positive role model in promoting Care of Older People as a future career. Teaching and training play an important part in encouraging HCPs into specialised training. Simulation is an important area to supplement training that guides learners through an experience in a controlled, safe environment [7]. Simulation has a number of benefits: facilitators have control over the learning tasks, and they will support and guide learners who have the opportunity to practice clinical skills and human factor skills safely with timely feedback in a post-simulation debrief [8]. Simulation training in medical education is supported by the Joint Royal Colleges of Physicians Training Board (JRCPTB), who value the use of simulation techniques to enhance training [9]. As a result of the supporting evidence towards simulation, it is now present in most medical specialities and nursing curricula at undergraduate and postgraduate levels [10].

### How do we improve attitudes towards older people?

We can engage students with a variety of learning techniques in clinical and non-clinical settings, such as simulation-based education (SBE). Simulation technologies include role-play, simulated participants, full body manikins, part task manikins and more computer-based simulation (e.g. virtual reality, augmented reality). Ageing simulation where students don tan ageing suit or equipment including virtual reality that can mimic the effects of ageing gives the learner the opportunity to experience hands on what it could feel like to be an older person with co-morbidty in a safe, controlled environment. Given the increasing interest in this area, and the need to support such teaching practice by evidence, there is a need to specifically understand this emerging area. The pivotal point to this simulation is for the student to then complete activities of daily living (ADL) to appreciate how the suit affects their ability to perform basic everyday tasks. In relation to attitudes, this has the potential to make an impact on empathy towards patients suffering chronic ill health.

The aim of this scoping review is to determine what is known about simulating ageing experiences on HCP students and the potential impact on their attitudes towards older people.

## Methods

### Methodological approach to literature review

Given the emerging use of simulating ageing experiences, we chose to conduct a scoping literature review to map what is known in the literature in simulating ageing experiences for HCP students and its impact on attitudes towards older patients. With this approach, we aimed to map the body of evidence base around this subject, assess its relevance in older patient’s care and identify important gaps in the literature.

### Research team

Our research team included individuals from various backgrounds: a Consultant Geriatrician (EN), an Academic Clinical Lecturer (ADS) and a Clinical Professor of Simulation and GP (GJG).

### Scoping review framework

We applied Arksey and O’Malley’s framework to our scoping literate review; see Appendix 1 [11]. This comprised the first five stages. We did not include the optional consultation exercise (Stage 6) due to pragmatic considerations of time limitation, resource constraints and the COVID 19 pandemic.

#### Stage 1: Defining the review question

We applied the ‘Population, Situation’ tool to establish our scoping review question [12]. The *Population* was HCP students and the *Situation* ageing simulation and its impact on attitudes towards older patients. Our main objectives were:

- To review the methods of simulation interventions used to create the simulation experience.
- To consider the outcome measures of the simulation experience, the impact this had on HCP students and their attitudes towards older patients.

#### Stages 2 and 3: Search strategy and selection criteria

**Figure 1:**
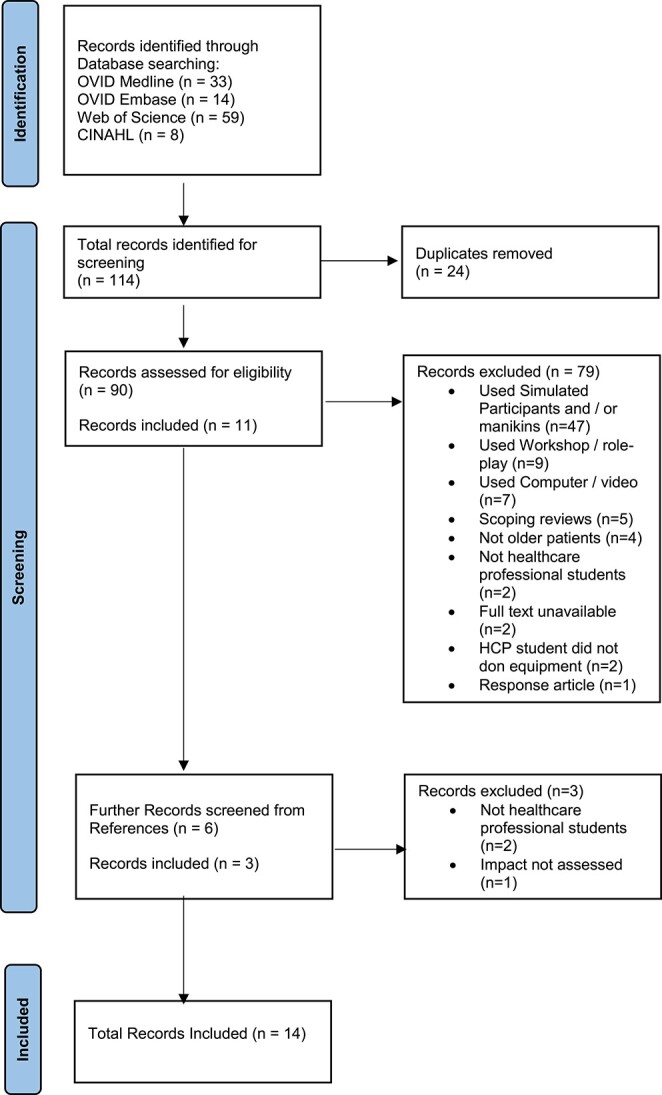
PRISMA flowchart of the screening and selection process

Please refer to Appendix 2. The full search strategy used for all electronic databases can be found in Tables 1–4. Our inclusion and exclusion criteria can be found in Appendix 3. Our Preferred Reporting Items for Systematic Reviews and Meta-Analyses (PRISMA) flowchart (Figure 1) illustrates our screening and selection process resulting in 14 included studies.

**Table 1:** Search terms used for MEDLINE

Set	Search statement
1	Patient simulation or simulation training/
2	Ag* simulation suit*.mp.
3	Ag* simulation experience*.mp.
4	Students, health occupations/ or students, nursing or students, medical/
5	Education, medical, undergraduate/ or health professional undergraduate.mp.
6	Undergraduate medical student*.mp.
7	Undergraduate nursing student*.mp.
8	Undergraduate health professional student*.mp.
9	Geriatric assessment/ or geriatrics or health services for the aged or geriatric medicine.mp.
10	Geriatric nursing or elderly care.mp.
11	Care of the elderly.mp.
12	1 or 2 or 3
13	4 or 5 or 6 or 7 or 8
14	9 or 10 or 11
15	12 and 13 and 14
16	Limit 15 to (English language and humans)

**Table 2:** Search terms used for Embase

Set	Search statement
1	Patient simulation/
2	Simulation training/
3	Ag* simulation suit*.mp.
4	Ag* simulation experience*.mp.
5	Nursing student/ or medical student/
6	Students health occupations.mp.
7	Education medical undergraduate.mp.
8	Health professional undergraduate.mp.
9	Undergraduate medical student*.mp.
10	Undergraduate nursing student*.mp.
11	Undergraduate health professional student*.mp.
12	Geriatrics/ or geriatric assessment/
13	Health services for the aged.mp.
14	Geriatric medicine.mp.
15	Geriatric nursing.mp.
16	Elderly care/
17	Care of the elderly.mp.
18	1 or 2 or 3 or 4
19	5 or 6 or 7 or 8 or 9 or 10 or 11
20	12 or 13 or 14 or 15 or 16 or 17
21	18 and 19 and 20

**Table 3:** Search terms used for Web of Science

Search topic	Search terms
1	Patient simulation or ‘simulation training’ or ‘Ag$ simulation suit*’ or ‘Ag$* simulation experience*’
2	Student medical or students nursing or students health occupations or education medical undergraduate or Health professional undergraduate or undergraduate medical student* or undergraduate nursing student* or undergraduate health professional student*
3	Geriatrics or geriatric assessment or health services for the aged or geriatric medicine or geriatric nursing or elderly care or care of the elderly
	#1 AND #2 AND #3 and English (languages)

**Table 4:** Search terms used for CINAHL

Search ID#	Search terms
S1	(MH ‘patient simulation’)
S2	‘Simulation training’
S3	‘Ag* simulation suit*’
S4	‘Ag* simulation experience*’
S5	(MH ‘students, nursing’)
S6	(MH ‘students, allied health’)
S7	(MH ‘geriatrics’) or (MH ‘geriatric assessment’)
S8	(MH ‘health services for the aged’)
S9	(MH ‘gerontologic nursing’)
S10	‘Elderly care’
S11	‘Care of the elderly’
S12	S1 OR S2 OR S3 OR S4
S13	S5 OR S6
S14	S7 OR S8 OR S9 OR S10 OR S11
S15	S12 AND S13 AND S14

#### Stage 4: Charting the data

EN constructed a data extraction table on Microsoft Excel® for the 14 included studies, found in Appendix 3. Extracted data were in the following broader categories: study details, the simulation intervention, impact and learning. Relevant study details extracted included authors, year of publication, location of study and country of publication. The type of study was noted along with keywords used, HCP students involved, number of students and year of study. Lastly, the aim of the study was documented. The simulation intervention included the sensory and functional disabilities created, equipment used and ADLs completed. We documented the simulation event duration, method of data collection used and length of follow-up, if applicable. To conclude, we examined impact and learning, comprising of outcome measures, impact on HCP students, relevance to older people care and learning for the future.

#### Stage 5: Collating, summarising and reporting the results

Microsoft Excel® was used to complete basic numerical analysis of the included studies. Given the diversity of the included studies, we have outlined relevant themes, through a thematic analytical approach, from the charted data within our reported results and our interpretation of the findings within our discussion. The thematic analytical approach was a collective, inductive and iterative process until a consensus was obtained by all researchers that the constructed themes represented the studies in our scoping review and addressed our research question. Moreover, researchers were collectively reflexive throughout this process.

## Results

### Publication characteristics

For publication characteristics please refer to our supplementary data.

### The simulation experience

All studies involved the donning of simulation equipment and immersion into tasks to complete basic and instrumental ADLs [13–26]. There were two distinct styles of simulation. Firstly, workshop based simulation stations where students don simulation equipment and complete ADL tasks. These either are stand-alone simulation activities or included as part of a larger geriatric module alongside traditional lectures, large group teaching and small group discussion. Secondly, Ageing Games are simulated learning activities, which involve structured role-play where the students adopt the identity of or ‘becomes’ an older person. Described by Chen *et al.* students acquire simulated physical, sensory and cognitive deficits by donning simulation equipment as they move through the levels with each level resulting in more disability and loss of independence [16]. Some games like the Geriatric Medication Game also have psychological and financial problems added and managing medications. They are tasked to complete ADLs throughout the levels which may include scenarios based around home life, shopping or healthcare services [16]. With both interventions, the trainers facilitate an introductory pre-brief session and post-intervention debrief discussion.

### Methods used to create the simulation experience

#### Sensory impairments

All studies simulated visual impairment [13–26]. Glasses or safety goggles were modified by smearing petroleum over them or applying clear tape to manufactured glasses simulating a range of visual disturbances like macular degeneration, cataracts and diabetic retinopathy. Twelve studies simulated hearing impairment with earplugs or earmuffs [13–18, 20, 21, 23–26]. Some used headphones playing distracting background audio [13, 15]. This sound distortion simulates what it might be like for a person who has moderate to severe dementia. One study included thick gloves to reduce hot and cold sensation and a nose plug to mimic loss of smell [21].

#### Mobility issues

Thirteen of the 14 studies simulated mobility issues and/or joint dexterity [13–21, 23–26). This ranged from simulating osteoarthritis with joint stiffness and restriction alongside arthritic pain. Studies were quite ingenious mimicking foot pain with popcorn kernels [17, 18, 26] or macaroni [19] inside socks or shoes. The use of weights over the chest, wrists and ankles to mimic loss of muscle strength leading to fatigue [15, 19–21, 23]. Kyphosis was created with a type of back protector or hanging weights from the shoulders [19, 20, 23]. Gloves were used to reduce hand dexterity and loss of sensation; some taped or sewed the gloved fingers together [13, 14, 17, 19, 21, 23, 25]. One study simulated neuropathy in the feet with foam-filled slippers [19].

#### Specific illnesses

Thirteen studies simulated a combination of visual impairment and/or hearing impairment and/or mobility issues [13–21, 23–26]. Other conditions, disabilities and impairments were simulated included hemiparesis after a stroke [19], shortness of breath using a corset [21] and Parkinsonian gait by tying twine around the ankles [19].

### ADLs simulated

A varied number of ADLs were simulated within studies, ranging from one to five. There is obvious difficulty simulating toileting, continence and personal care given how students would feel attempting these with peers and teachers present. One study looked at how students attempted the initial ADL task then they were given an adaption to make the task easier [25]. From our scoping review, managing medications, managing finances and functional mobility were the most commonly simulated ADLs.

### Outcome measures and follow-up

In terms of methods of data collection, three of the fourteen studies used post-test questionnaires [18, 23, 24]. The other eleven used pre-test and post-test questionnaires [13–17, 19–22, 25, 26]. Two studies achieved qualitative data from semi-structured interviews and open-ended questions [20, 21]. Four studies had some longer-term follow-up. Torkshavand *et al*. [13] completed a post-test at 1 week and the follow-up at 1 month. Pacala *et al*. completed their post-test evaluation either at the end of the session or at the end of the clerkship [18] or 1 to 2 weeks following the simulation experience [26]. Koh *et al.* [22] completed their pre-test at the start of their module and a post-test 2 weeks after the end of the module. Likert Scales were used to gain basic quantitative data. A number of different scales were used to collect data around attitudes and empathy, a number are described in the section regarding impact on HCP students’ attitudes towards older people. Others scales used can be found in Appendix 4.

### Impact on HCP students

In the majority of our included studies simulation resulted in positive improvements in medical students’ knowledge of Care of Older People and nursing students’ knowledge of Care of Older People nursing [13, 14, 16–24, 26]. Students gained an insight into the awareness of Care of Older People as a specialty and the skills required to approach an older person [13, 17, 20–22, 26]. There was greater understanding of older people in general and the challenges they potentially face with ageing [14, 16, 19].

The simulation intervention had an impact on the HCP students as a mode of learning [18]. Largely the simulation interventions received positive feedback being scored as very good or excellent on Likert scales [18, 19, 23–26]. Students felt these simulation sessions should be integrated into their curriculums [14], would recommend them to fellow students [23] and would volunteer for sessions if they were not mandatory [17]. From qualitative comments, there was a level of entertainment and enjoyment to these sessions [21]. They were felt to be of educational value [18] with documented effect that the session would change students’ behaviours in future interactions with older people [14].

### Impact of HCP students’ attitudes towards older people

In general, attitudes towards older people improved [14, 17–22, 26] as did empathy towards caring for older people and/or carers [17, 18, 20, 21, 26]. One study by Lucchetti *et al*. [15] found mixed results. Their study was on an Ageing Game with half of students in the intervention group that donned the simulation equipment and the other half in a non-intervention group. They measured attitudes towards older people using the University of California Los Angeles Geriatrics Attitudes Scale [27]. They determined attitude and empathy using the Maxwell–Sullivan Attitudes Scale [28]. Knowledge on facts and positive views about ageing were assessed by Palmore [29]. They found the intervention group was associated with an improvement in empathy but worsening of attitude while the non-intervention group was associated with an improved attitude overall and a positive view about ageing but no change in empathy [15]. This mixed effect led to the researchers considering combining the workshops with the overall aim of improving attitude and empathy towards older people.

Another study by Robinson *et al*. used the Ageing Semantic Differential [25]. This measures attitudes; quantifies bias and negative stereotypes towards older people [30]. This study found an overall negative attitude towards older people however; there was a highly significant change in the instrumental-ineffective domain of the Ageing Semantic Differential, the most dominant of three dimensions within this scale. Scoring highly in this domain demonstrates one as being: capable of actively pursuing goals, adaptive to change and suited for ‘being where the action is’ [30]. Therefore, even though the outcome was negative, there is a positive aspect that these students can adapt and their attitudes can change.

Negative emotions were experienced by students [16, 19–21, 23, 26]. Mentioned most frequently was frustration [16, 19, 23, 26] at the length of time they had to queue or wait in an ageing game or that they could not complete a normally easy task. A basic emotion expressed was anger together with annoyance and impatience [16, 19]. Some students voiced feelings of paranoia, anxiety, apprehension, fear and embarrassment when wearing the equipment and taking part in the intervention [19, 20]. Regarding the future and consequences of ageing, a number of students felt depressed or had feelings of helplessness and desperateness [19, 20]. Tremayne *et al*. [23] during their role-play simulation had two students wear a simulation suit at the same time with the other students in a caregiver role. Qualitative feedback from students who wore the suit identified that they felt their peers had treated them differently and lacked insight into how difficult it was to complete the ADL tasks [23]. Sorrow was recorded both in a past and future tense with students feeling sadness in the past on how they had felt about or had previously treated older people [21]. There was also sadness for the future that these illnesses and physical impairments could be a future reality [21]. These negative attitudes may influence a HCP students’ approach to an older person and lead to ageism. As physicians working in Care of Older People, we need to promote positive attitudes towards older people and healthy successful ageing.

## Discussion

This scoping review provides, from the evidence base, an overview of the different methods used to simulate what it might feel like to be an older person with disability and specific illnesses associated with older age.

Students immersed themselves in ADL tasks with the intention to provide an understanding of challenges that an older person may encounter. We have found an array of different approaches to simulation equipment ranging from the use of basic goggles and earplugs to commercially available, complex ageing-suits. The intervention itself and setting of the simulation sessions differ, from locally created workshops to the use of a licensed Geriatric Medication Game.

We reviewed what HCP students have learnt from these simulation experiences. This includes gaining knowledge in the speciality of Care of Older People, in particular the skills and awareness required for the clinical approach to an older person, who often has multi-morbidity, frailty, cognitive decline, sensory impairments, mobility issues and polypharmacy. We also outlined how students’ attitudes towards older people changed because of the simulation experience with themes around empathy, caring and emotions. We acknowledge that not all outcomes were positive. As discussed two studies had mixed results and there were negative emotions described by students during feedback and debrief sessions. These are an important and integral part of the learning experience. SBE may not be a mode of learning appreciated by all students. However, the vast majority of studies show positive evidence supporting SBE as shown in the JRCPTB literature review for Core Medical Training [9]. They identified 95 papers that used SBE in teaching content relevant to General Internal Medicine. Positive evidence to support SBE was found in 90 studies, neutral evidence in 4 studies and one study by Curtis *et al*. found negative effects of teaching relevant to ‘communication in a consultation’ about end-of-life care [31]. Simulation experiences however can always be improved. Methods of ensuring successful simulation are to set clear objectives, formal post-simulation evaluation by learners with the results acted upon to continuously improve and optimise the simulation experience, sufficient time for feedback and debriefing with a focus on human factors and regular evaluation of performance [10].

### Relevance to geriatrics

With growing numbers of persons of older age, there will be increasing demand for healthcare within the Care of Older People specialty. Our review highlights several areas, which should receive a focus in medical, nursing, pharmacy and AHP curricula, such as teaching about ageing in a positive way with the promotion of *successful* ageing [32]. Excellent communication skills with older people, family and carers is vital as we strive for patient-centred care. Poor quality patient-carer interactions have a negative impact on HCPs attitudes towards older service users, whether explicit or implicit [33]. Negative perceptions of ageing and attitudes to age can create psychological barriers in older people towards rehabilitation, motivation and treatment response [33]. Training HCPs on the effects of ageism in clinical and care settings should include raising awareness of ageism, how it affects older patients and how access to treatments can be threatened by negative age stereotypes [34].

### Cost impact

Only one study by Pacala *et al.* [18] looked at the cost implications of this type of simulation experience. They reflected on 10 years of conducting the Ageing Game and if it was value-for-money. They examined the supplies required for each of three stations, the personnel, finding a considerable number of props were donated, and facilitators worked as unpaid volunteers. The initial starting costs were $1,059 (£866.22 at the time of writing this scoping review), with subsequent games costing $529 (£432.70) each, used to cover personnel costs, as props could be re-used. The team felt the direct cost for materials were trivial provided the workshop was repeated on a regular basis. The major cost was faculty and staff time with the challenge being to maintain the energy and enthusiasm of faculty during what can be a repetitive educational activity. With this in mind, the team felt that the recruitment of more volunteers knowledgeable in geriatrics could make the Ageing Game more successful and cost-efficient.

From our scoping review, this has shown that low-fidelity simulation equipment can be used just as effectively as a high-fidelity manufactured suit. There was a resourceful approach to simulation equipment design to recreate impairments with simple low-cost materials e.g. popcorn kernels and macaroni, simple bandages and twine. This has the obvious financial benefit as well as allowing the development of more reproducible techniques that can then be cascaded to resource-poor environments given the rapid changes in population demographics in less economically developed countries.

### Main gaps in the literature

The majority of studies contained either medical or nursing students whereas, in reality, we do not work in such uniprofessional silos. Potential approaches include team working and interprofessional education between all specialities of HCP students with an introduction to Care of Older People at the beginning of their undergraduate education and throughout training. This foundation could then be developed in the postgraduate era to create a continuum between these two levels of education with the intention to weave interprofessional education into continuing professional development [35]. Positive effects include improvement in students’ attitudes and perceptions of one another and increases in collaborative knowledge and skills [36]. However the longitudinal benefits of these positive effects are uncertain and worthy of future research.

### Limitations

Despite the novelty of this scoping review, it has to be considered within its limitations. Our search strategy of four databases was robust, however, as with any review; articles satisfying the research criteria may have been unintentionally omitted.

We excluded studies that did not centre on older people and the simulation of ageing. Studies were omitted if the HCP students did not don the equipment themselves, as we wanted to assess how the experience affected them and changed their attitudes towards older people. This included virtual reality. Research of virtual reality ageing simulations would be worthy of future research. As clinicians working in medicine, we were keen to focus on studies relevant to our clinical setting, hence why we excluded dentistry students.

Grey literature and non-academic articles were excluded. All articles were in English, we did not search for articles in non-English languages. We did not conduct the optional stage 6 consultation exercise of scoping review methodology as per Arksey and O’Malley [11] due to practical considerations of time limitation and resource constraints. Scoping reviews do not assess for quality of the students included. Nonetheless, this has provided a good platform for future literature reviews that do consider quality of studies (e.g. systematic review).

### Recommendations

We have a number of recommendations from this scoping review.

Firstly, interprofessional education represents a significant gap and future research integrating interdisciplinary HCP students in an ageing simulation experience is needed.

Secondly, there is a need to build on successful ageing and changes to students’ attitudes towards older people. Several studies commented about supporting post-simulation with discussions and presentations [19, 22, 24–26]. This post-simulation debrief stage is an important learning conversation, with opportunities to micro-teach and consider positive outcomes while in that immersive environment.

Thirdly, we need to introduce HCP students to older people and Care of Older People medicine from the outset of training and learn as a continuum throughout undergraduate education and beyond. There is an opportunity to analyse students at different levels of training and consider longitudinal retention of learning.

## Conclusion

The use of simulation is an effective teaching technique used within Care of Older People [37]. Simulation is becoming routine within medical education and a way to engage learners in achieving their curricula goals [9]. The use of ageing equipment and suits can help replicate what it might feel like to have disabilities and co-morbidities associated with ageing. This gives HCP students the opportunity for experiential learning, while also improving empathy towards older people [15–17, 20, 26]. The use of ageing simulation as part of a Care of Older People attachment or module helps to raises awareness with the aim of inspiring and attracting HCP students into the specialty [18, 21]. As such, we suggest Care of Older People training should be introduced at the beginning of undergraduate HCP training and continue throughout HCP curricula, with particular focus on interprofessional education.

## Supplementary Material

aa-23-0315-File002_afad235
