# Supplementary material for: Stepping into the shoes of older people: a scoping review of simulating ageing experiences for healthcare professional students
Source: Age Ageing. 2023 Dec 28;52(12):afad235. doi: 10.1093/ageing/afad235 (PMC11025377; doi:10.1093/ageing/afad235)
Supplement: aa-23-0315-File002_afad235 [file aa-23-0315-file002_afad235.docx]

**Supplementary data**

Contents

Appendix 1…………………………………………………………………….Arksey and O’Malley’s framework

Appendix 2…………………….....................Stage 2 and 3: Search strategy and selection criteria

Appendix 3…………………………………………………………………………Inclusion and exclusion criteria

Appendix 4……………………………………………………………………………….Publication Characteristics

Appendix 5……………………………………………………………………………………….Data extraction table

Appendix 6………………Different scales used to measure attitudes, knowledge and empathy

| Appendix 1: Arksey and O’Malley’s framework | |
| --- | --- |
| Stage 1 | Identifying the research question |
| Stage 2 | Identifying the relevant studies |
| Stage 3 | Study selection |
| Stage 4 | Charting the data |
| Stage 5 | Collating, summarising and reporting the results |
| Stage 6 | Consultation exercise (optional) |

Reference:

1. Arksey H, O’Malley L. Scoping studies: towards a methodological framework. International Journal of Social Research Methodology 2005;8:19–32.

Appendix 2: Stage 2 and 3: Search strategy and selection criteria

A pilot search was completed using Ovid MEDLINE (US National Library of Medicine, Bethesda, Maryland, USA); this included the search of three concepts: the simulation experience, HCP students and older people care. The full search strategy used for all electronic databases can be found in Tables 1-4. On the 30^th^ December 2021, four electronic databases were searched: MEDLINE, EMBASE (Reed Elsevier PLC, Amsterdam, the Netherlands), Web of Science (Thomson Reuters, USA) and Cumulative Index to Nursing and Allied Health Literature (CINAHL, EBSCO, Ipswich, Massachusetts, USA). We maintained a broad range of article types to ensure we included all appropriate sources of knowledge. This comprised of empirical-based research, book chapters, conference presentations and editorials. Our search accumulated in 114 citations for review, 24 duplicates were removed. This left 90 studies whose titles and abstracts were independently screened by two members of the research team (EN and ADS). Any title and abstract of papers that were felt to be relevant had their full-texts obtained. Individual studies were then assessed against our inclusion and exclusion criteria, this table can be found in Appendix 2. We did not put any time restriction onto the searches and we only included studies that were written in English. Seventy-nine studies were excluded as they were felt to be out with scope. We screened for further references by reviewing the bibliographies of relevant studies. Any conflicts regarding inclusion of a particular study were sent for third party review to GJG. Fourteen included studies, which satisfied the study criteria, remained. Our Preferred Reporting Items for Systematic Reviews and Meta-Analyses (PRISMA) flowchart (Figure 1) illustrates our screening and selection process. As in keeping with scoping review methodology, studies were not for assessed for quality [11].

Reference:

1. Arksey H, O’Malley L. Scoping studies: towards a methodological framework. International Journal of Social Research Methodology 2005;8:19–32.

Appendix 3: Inclusion and exclusion criteria

| **Inclusion and exclusion criteria** | |
| --- | --- |
| **Inclusion criteria** | All studies that involve donning a piece or pieces of simulation equipment / suit.  HCP students including medical, nursing, AHPs and pharmacy.  HCP students don the simulation equipment / suit.  Studies involved in Care of Older people.  Studies in the English language |
| **Exclusion criteria** | All studies that involve simulation as an interaction without donning a piece(s) of simulation equipment / suit i.e. simulated participants, manikins, role play, computer-based etc.  HCP students in dentistry.  HCP students involved in the study but do not don the simulation equipment / suit.  Studies not involved in Care of Older people.  Studies not in the English language.  If the full text of the study was unavailable.  Scoping reviews.  If the impact of the study on student’s attitudes towards older people was not assessed. |

Appendix 4: Publication Characteristics

The 14 included studies were by 13 different authors [13-26]. Studies were published between 1989 to 2021; we did not exclude any based-on time since publication. Seven (50%) studies were published after 2012, within 10 years of this review. Eight studies (58%) had their country of origin in the United States of America; two studies (14%) were based in Turkiye (Turkey) and one study (7%) from each of Brazil, Iran, Singapore and the United Kingdom. The type of study varied with seven (50%) being a mixed-methods model, five (36%) quasi-experimental, one (7%) quantitative and one (7%) qualitative. The studies included HCP students from multiple disciplines: eight (58%) studies involved medical students; five (36%) involved nursing students. One study by de Abreu et al included students from different HCP disciplines including psychiatry, medicine, physician assistant and pharmacy [14]. The year of study for undergraduates who participated varied: five (36%) studies involved first year students; two (14%) with second year students; five (36%) with both third or fourth year; and one (7%) of final year students. The de Abreu study, mentioned above, had different HCPs and mixed years of study [14]. The length of the simulation intervention ranged from 10 minutes to three hours. No length of time was given for the intervention in three studies. The average time for the other 11 studies was 108 minutes.

References:

1. Torkshavand G, Khatiban M, Soltanian A. Simulation-based learning to enhance students’ knowledge and skills in educating older patients. Nurse Education in Practice 2020;42:102678.
2. de Abreu I, Hinojosa-Lindsey M, Asghar-Ali A. A Simulation Exercise to Raise Learners’ Awareness of the Physical and Cognitive Changes in Older Adults. Acad Psychiatry 2017;41:684–7.
3. Lucchetti A, Lucchetti G, de Oliveira I et al. Experiencing aging or demystifying myths? – impact of different “geriatrics and gerontology” teaching strategies in first year medical students. BMC Med Educ 2017;17, DOI: 10.1186/s12909-017-0872-9.
4. Chen A, Kiersma M, Yehle K et al. Impact of the Geriatric Medication Game® on nursing students’ empathy and attitudes toward older adults. Nurse Education Today 2015;35:38–43.
5. Varkey P, Chutka D, Lesnick T. The Aging Game: Improving Medical Students’ Attitudes Toward Caring for the Elderly. Journal of the American Medical Directors Association 2006;7:224–9.
6. Pacala J, Boult C, Hepburn K. Ten Years’ Experience Conducting the Aging Game Workshop: Was It Worth It? Journal of the American Geriatrics Society 2006;54:144–9.
7. Lorraine V, Allen S, Lockett A et al. (1998) “Sensitizing students to functional limitations in the elderly: an aging simulation.” *Family Medicine*, 30(1), pp. 15–18.
8. Sari D, Taskiran N, Baysal E et al. Effect of an aged simulation suit on nursing students’ attitudes and empathy. Eur Geriatr Med 2020;11:667–75.
9. Demirtas A, Basak T. Daily life activities simulation: Improving nursing students’ attitudes toward older patients. Jpn J Nurs Sci 2020;18, DOI: 10.1111/jjns.12375.
10. Koh G, Merchant R, Wee L et al. (2012) “The knowledge-attitude dissociation in geriatric education: Can it be overcome? ” *Annals of the Academy of Medicine, Singapore*, 41(9), pp. 383–389. Available at: https://doi.org/10.47102/annals-acadmedsg.v41n9p383.
11. Tremayne P, Burdett J, Utecht C. Simulation suit aids tailored care. Nursing Older People 2011;23:19–22.
12. McVey L, David D, Cohen H. The ‘Aging Game’. JAMA 1989;262:1507.
13. Robinson S, Rosher R. Effect of the “Half-Full Aging Simulation Experience” on Medical Students’ Attitudes. Gerontology &amp; Geriatrics Education 2001;21:3–12.
14. Pacala J, Boult C, Bland C et al. Aging Game Improves Medical Students’ Attitudes Toward Caring for Elders. Gerontology &amp; Geriatrics Education 1995;15:45–57.

Appendix 5: Data extraction table

| **Study details** | | | | | | | | |
| --- | --- | --- | --- | --- | --- | --- | --- | --- |
| **No.** | **Author/Reference** | **Year** | **Location** | **Study methods** | **Participant profession** | **Aims** | **Cohort size** | **Year of learner** |
| 1 | Torkshavand et al.  Torkshavand G, Khatiban M, Soltanian A. Simulation-based learning to enhance students’ knowledge and skills in educating older patients. Nurse Education in Practice 2020;42:102678. | 2020 | Iran | Quasi-experimental design | Nursing | To improve knowledge, attitudes and skills towards older patients | 70  (35 in each group) | Third |
| 2 | de Abreu et al.  de Abreu I, Hinojosa-Lindsey M, Asghar-Ali A. A Simulation Exercise to Raise Learners’ Awareness of the Physical and Cognitive Changes in Older Adults. Acad Psychiatry 2017;41:684–7. | 2017 | USA | Quantitative pretest-posttest design | 47% psychiatry residents 43% medical students 8% physician assistant students 2% pharmacists | To raise learners' awareness of, and attentiveness to, physical and cognitive changes experienced by the aging population | 51 49 participated in study 48 completed both pre- and posttests | Mixed |
| 3 | Lucchetti et al.  Lucchetti A, Lucchetti G, de Oliveira I et al. Experiencing aging or demystifying myths? – impact of different “geriatrics and gerontology” teaching strategies in first year medical students. BMC Med Educ 2017;17, DOI: 10.1186/s12909-017-0872-9. | 2017 | Brazil | Intervention-based quasi-experimental | Medical | Determine the impact of two educational strategies, Experiencing Aging (EA) and Myths of Aging (MA) compared to a control group on attitudes, empathy and knowledge | 230 72 in control group 82 in EA Simulation 76 in MA workshop | First |
| 4 | Chen et al.  Chen A, Kiersma M, Yehle K et al. Impact of the Geriatric Medication Game® on nursing students’ empathy and attitudes toward older adults. Nurse Education Today 2015;35:38–43. | 2015 | USA | Quasi-experimental pretest-posttest design | Nursing | Impact of aging simulation game on empathy and attitudes towards older adults and understanding of patients' experiences in healthcare system | 58 | First |
| 5 | Varkey et al.  Varkey P, Chutka D, Lesnick T. The Aging Game: Improving Medical Students’ Attitudes Toward Caring for the Elderly. Journal of the American Medical Directors Association 2006;7:224–9. | 2006 | USA | Mixed-methods | Medical | To evaluate the effectiveness of a modified aging game to enhance medical students' attitudes towards caring for elderly patients and improve general attitudes toward the elderly | 84 | First |
| 6 | Pacala et al.  Pacala J, Boult C, Hepburn K. Ten Years’ Experience Conducting the Aging Game Workshop: Was It Worth It? Journal of the American Geriatrics Society 2006;54:144–9. | 2006 | USA | Mixed-methods | Medical | To sensitise learners to the experience of aging with disability and to promote self-reflection about attitudes caring for older persons. | Approximately 1500 over 10 years 673 evaluations 477 written comments | Third/  fourth year |
| 7 | Lorraine et al.  Lorraine V, Allen S, Lockett A et al. (1998) “Sensitizing students to functional limitations in the elderly: an aging simulation.” *Family Medicine*, 30(1), pp. 15–18. | 1998 | USA | Mixed-methods | Medical | To experience the physical frailties of aging, develop creative problem-solving techniques, identify feelings regarding the experience of functional loss and develop proactive clinical approaches to the care of the elderly | 100 | Fourth |
| 8 | Sari et al.  Sari D, Taskiran N, Baysal E et al. Effect of an aged simulation suit on nursing students’ attitudes and empathy. Eur Geriatr Med 2020;11:667–75. | 2020 | Turkey | Mixed-methods | Nursing | To explore the effect of use of an age simulation suit on empathy and attitudes of nursing students towards older persons | 260 completed pre-simulation tests. Students divided into high, medium, low groups. 10 students randomised from each group. Study group = 30 | "Senior" |
| 9 | Demirtas & Basak  Demirtas A, Basak T. Daily life activities simulation: Improving nursing students’ attitudes toward older patients. Jpn J Nurs Sci 2020;18, DOI: 10.1111/jjns.12375. | 2021 | Turkey | Quasi-experimental pretest-posttest design | Nursing | Evaluate the effects of daily life activities simulation on the attitudes of students towards older adults | 161 eligible to participate. 119 agreed to participate. Control group n=60. Simulation group n=59. | First |
| 10 | Koh et al.  Koh G, Merchant R, Wee L et al. (2012) “The knowledge-attitude dissociation in geriatric education: Can it be overcome? ” *Annals of the Academy of Medicine, Singapore*, 41(9), pp. 383–389. Available at: https://doi.org/10.47102/annals-acadmedsg.v41n9p383 | 2012 | Singapore | Quasi-experimental pretest-posttest design | Medical | Determine whether a holistic education programme incorporating multiple educational strategies such as early exposure, ageing simulation and small group teaching results in improving geriatric knowledge and attitudes  The module aims to provide the foundation for competent and compassionate care of older adults through didactic and experiential components  The ageing simulation workshops aim to sensitise students to the process of ageing | Baseline cohort n=254. 195 (76.8%) completed UCLA-GAS.  189 completed UCLA-GKT. Intervention cohort n=261. 204 completed pre-test. 169 completed post-test UCLA-GKT 198 completed post-test UCLA-GAS | Second |
| 11 | Tremayne et al.  Tremayne P, Burdett J, Utecht C. Simulation suit aids tailored care. Nursing Older People 2011;23:19–22. | 2011 | UK | Mixed-methods | Nursing | Evaluate the effectiveness of an aged simulation suit in pre-registration nurse education to enhance knowledge and understanding of some of the changes that can occur as a consequence of ageing | 90 students completed the evaluation. 10 to 15 students per session. 2 students volunteer to wear the suit. | Second |
| 12 | McVey et al.  McVey L, David D, Cohen H. The ‘Aging Game’. JAMA 1989;262:1507. | 1989 | USA | Qualitative | Medical | To stimulate students about their beliefs and values about old age and sensitise them to the problems facing the elderly | 112 15-20 participants per game. | First year |
| 13 | Robinson & Rosher  Robinson S, Rosher R. Effect of the “Half-Full Aging Simulation Experience” on Medical Students’ Attitudes. Gerontology &amp; Geriatrics Education 2001;21:3–12. | 2001 | USA | Mixed-methods | Medical | To allow students to experience functional decline that may occur with normal aging while learning how function can be improved by simple environmental adaptions. | 49 | Third |
| 14 | Pacala et al.  Pacala J, Boult C, Bland C et al. Aging Game Improves Medical Students’ Attitudes Toward Caring for Elders. Gerontology &amp; Geriatrics Education 1995;15:45–57. | 1995 | USA | Mixed-methods | Medical | Evaluate the effectiveness of a modified version of the Aging Game on students' empathy, attitudes, knowledge of geriatrics and beliefs about elderly persons | 71 students enrolled in course. 55 completed pre and post-tests. 39 students elected to participate. 10-20 in each workshop. | Fourth |

| **Simulation Intervention** | | | | | | | | | | | | | |
| --- | --- | --- | --- | --- | --- | --- | --- | --- | --- | --- | --- | --- | --- |
| **No.** | **Author** | **Simulation intervention** | **Simulation experienced** | **Equipment** | | **ADLs simulated** | | | **Duration** | | **Data collection method** | | **Follow-up** |
| 1 | Torkshavand et al. | Simulation-based learning (SBL) versus Lecture-based learning (LBL) | Hearing loss Visual impairment Joint stiffness | Ear muffs Modified safety eyeglasses Elbow and wrist supports Half-finger protecting gloves Firm foam cervical collar | | Managing medications | | | 20-25 minutes | | Locally developed pre and posttest questionnaires | | Post-test questionnaire within one week Follow-up questionnaire at one month |
| 2 | de Abreu et al. | Completion of 3 day-to-day activity tasks | Reduced visual acuity Limited manual dexterity Impaired hearing Cognitive impairment | Goggles Thick gloves Headphones playing distracting audio Complex, multistep directions | | Managing medications, communication and finances. Housekeeping and Home maintenance | | | 10 minutes | | Pre and posttest questionnaires | | n/a |
| 3 | Lucchetti et al. | Aging Game: EA group selected | Mobility issues Visual impairment Hearing impairment | Leg weights Modified glasses Headphones with background noise Cotton ear plugs | | Functional mobility | | | 1 hour | | Pre and posttest questionnaires | | n/a |
| 4 | Chen et al. | Aging game: Modified version of the Geriatric Medication Game | Mobility issues Dexterity loss Vision and hearing loss | Petroleum jelly-coated goggles  (not told what else) | | Dressing Housekeeping and Home maintenance | | | 3 hours | | Pre and posttest questionnaires | | n/a |
| 5 | Varkey et al. | Aging Game | Reduced manual dexterity Visual impairment Presbycusis Arthritis pain Pedal oedema Parkinsonian gait | Heavy rubber gloves Goggles with clear tape Foam earplugs Popcorn kernels in shoe Heavy athletic stockings on other leg  Tight elastic bandage around knees | | Managing medications Functional mobility Shopping  Managing finances | | | 3 hours | | Mixed Pre and posttest questionnaires | | n/a |
| **Simulation Intervention** | | | | | | | | | | | | | |
| **No.** | **Author** | **Simulation intervention** | **Simulation experienced** | **Equipment** | | **ADLs simulated** | | | **Duration** | | **Data collection method** | | **Follow-up** |
| 6 | Pacala et al. | Aging game  (Minnesota version) | Hearing loss Frozen shoulder Pain on walking Reduced mobility Visual impairment | Ear plugs Arm sling Athletic socks and popcorn Neck and leg brace Goggles | | Shopping Managing finances | | | "half-day" 30-45 minute introduction 3 stations (15-30 minutes per station) 30 minute post-simulation discussion | | Mixed Posttest evaluation Qualitative data obtained on strengths and weaknesses of workshop | | 10-year evaluation. Numeric evaluation completed immediately after the game or at the end of the clerkship |
| 7 | Lorraine et al. | Aging simulation exercise based in a retail store.  6 different medical conditions simulated then perform ADLs and iADLs. 3 phases: Pre-simulation discussion, Simulation and Post-simulation discussion | Parkinson's: Neck rigidity Shuffling gait Stooped posture Rheumatoid arthritis: Stiff fingers Pain on walking Stiff knees Diabetes: Visual loss Neuropathy hands and feet Stroke: Dominant-side weakness Visual changes Incontinence Glaucoma: Loss of peripheral vision | Cervical collar Ankles tied with twine Weight belts strapped to shoulders Taped fingers Macaroni in shoes Knee braces   Vaseline-coated glasses Gloves and foam-filled slippers  Arm sling  Glasses Disposable underwear Black glasses with pinholes | | Self-feeding Managing finances and medications Dressing Shopping | | | 3 hours | | Pre and posttest surveys | | n/a |
| **Simulation Intervention** | | | | | | | | | | | | | |
| **No.** | **Author** | **Simulation intervention** | **Simulation experienced** | **Equipment** | | **ADLs simulated** | | | **Duration** | | **Data collection method** | | **Follow-up** |
| 8 | Sari et al. | Students donned the suit then completed a series of ADLs | Sakamato Model M176 Visual Impairment Hearing loss Stooped posture Joint restriction Loss of muscle strength Reduced hand dexterity | Sakamato Model M176 Goggles Ear plugs Back protector Elbow and knee restrictors Wrist and ankle weights Gloves | | Functional Mobility Shopping | | | Not given | | Pre and post-simulation Basic Empathy Scale (BES) and Kogan's Attitude towards Old People Scale (KAOPS). Qualitative data obtained through semi-structured interviews and open-ended questions | | n/a |
| 9 | Demirtas & Basak | Control group received no intervention  Simulation group donned equipment then completed 4 stations of ADLs | Walking limitations Joint restriction SoB Cataracts Hearing loss Reduced hot/cold Loss of smell/taste | Ankle weights Bandages on hand, fingers, ankles Corset Glasses Ear plugs Thick gloves Eye bandage and nose plug | | Transferring / Functional Mobility Toileting Managing medications Dressing Self-feeding | | | 40-45 minutes | | Pretest-posttest | | n/a |
| 10 | Koh et al. | Module includes: Large group teaching Small group interactive workshops/tutorials which includes ageing simulation Small group discussion | Cataracts Peripheral neuropathy | Glasses Masking tape to tips of fingers | | Managing medications | | | Not told | | Baseline cohort 2nd year’s old curriculum 2009. Intervention cohort students’ new curriculum 2010. Pre-test first day of Geriatric Medicine module. Post-test 2 weeks after end of module | | n/a |
| **Simulation Intervention** | | | | | | | | | | | | | |
| **No.** | **Author** | **Simulation intervention** | **Simulation experienced** | **Equipment** | | **ADLs simulated** | | | **Duration** | | **Data collection method** | | **Follow-up** |
| 11 | Tremayne et al. | Session "physical and sensory understanding"  Role-play using a simulation suit | Aged simulation suit. (Japanese) Visual Impairment Hearing loss Stooped posture Joint restriction Loss of muscle strength Reduced hand dexterity | Aged simulation suit. (Japanese)  Goggles Ear plugs Back protector Elbow and knee restrictors Wrist and ankle weights Gloves | | Meal preparation Transferring / Functional mobility Toileting | | | 1 hour | | Post-test evaluation tool | | n/a |
| 12 | McVey et al. | The Aging Game 3 phases Phase I preparation Phase II the Aging Game Phase III post exercise discussion | Hearing loss Visual loss  Loss of mobility and independence | Ear plugs Safety goggles coated with petroleum jelly Hospital gown, bibs, physical restraint | | No ADLs formally tested | | | not told | | Postgame evaluation | | n/a |
| 13 | Robinson & Rosher | "Half-full" simulation experience First section: lecture Second section: simulation Students attempt task then repeat task with adaptation to make activity easier Third section: post-simulation discussion | Visual impairment Presbycussis Reduced hand dexterity Mobility issue | Glasses with yellow lenses Cotton balls Rubber gloves with fingers taped "Immobiliser" on one extremity | | Managing communication, medications and finances. Self-feeding Dressing | | | 3 hours | | Pre and post simulation | | n/a |
| **Simulation Intervention** | | | | | | | | | | | | | |
| **No.** | **Author** | **Simulation intervention** | **Simulation experienced** | | **Equipment** | | **ADLs simulated** | **Duration** | | **Data collection method** | | **Follow-up** | |
| 14 | Pacala et al. | Modified version of the Aging Game. 3 hour Elective workshop includes: 30-minutes introduction 90-minutes in the game 30-minutes post-exercise discussion 15-20 minutes unstructured interview with an elderly patient 5-minute musical videotape about a man's progression from childhood through old age | Hearing loss Pedal oedema Foot pain from arthritis Visual impairment Mobility issues Dependency in nursing home | Ear plugs Heavy socks Popcorn kernels in shoes Goggles Braces, slings Hospital gowns, "occasional restraint" | | "Students performed assigned instrumental ADLs in the simulated environments" | | | 3 hours workshop  90 minutes in the Aging game | | Pre and posttest evaluation | | Post-test 1 to 2 weeks following the exercise |

| **Impact and Learning** | | | | | |
| --- | --- | --- | --- | --- | --- |
| **No.** | **Author** | **Outcome measures** | **Impact** | **Relevance to Geriatrics** | **Learning for the future** |
| 1 | Torkshavand et al. | Los Angeles Geriatric Attitudes Scale | All students demonstrated improvement in knowledge and skills in older patient education. SBL group had larger and more durable improvement (all p's <0.001). | Focus on communication with older patients with COPD, how to use inhalers and teach effective breathing. | Nursing only, can be applied to other healthcare students. No longitudinal data. |
| 2 | de Abreu et al. | Approaches to Dementia Questionnaire (ADQ) 2 domains Hope and Person-centred | Statistically significant improvement in ADQ scores indicating an increase in positive attitudes. 96% students felt it would impact their practise behaviours. 100% indicated that the simulation should be implemented in the curriculum. | Leaners were in a core psychiatry rotation. Increasing need for geriatric services, few choosing to enter subspecialty geriatric psychiatry practice. Important that learners gain an appreciation of the experiences of older people. | Short time interval between tests. Lack of data examining the individual ADQ questions. No longitudinal data. Small sample size. Diverse learners at different levels of knowledge and experience. |
| 3 | Lucchetti et al. | Attitudes towards older persons assessed with UCLA attitudes and Maxwell-Sullivan. Empathy assessed with Maxwell-Sullivan. Knowledge on facts and positive view about aging Palmore. Cognitive knowledge | Mixed effect. EA intervention associated with an improvement in empathy but worsening of attitude. MA intervention associated with an improved attitude overall and positive view about aging but no change in empathy | During a first-year course called "Integrative themes in Clinical Practice" | Short time interval between tests (2 hours). No longitudinal data. Beginning of first year, compare between students with greater experience e.g. final years. Combine the workshops, MA then EA. Medical students only, one medical school. Could look at other students and different cultures. |
| 4 | Chen et al. | Kiersma-Chen Empathy Scale (KCES). Jefferson Scale of Empathy-Health Professions Students (JSE-HPS). Aging Simulation Experience Survey (ASES). | Empathy towards older adults significantly improved overall. Improvements seen in 7 out of 13 questions related to attitudes and healthcare understanding. At least 75% of students experienced annoyance, frustration and impatience during the GMG. | Nursing clinical course focusing on the care of older adults | Short time interval between tests. No longitudinal data. Small sample size, limited generalisability, 88% female students. Add to curriculum for reinforcement. Students self-reported empathy, should evaluate relationship between self-perception and observable measures from faculty/staff. |
| **Impact and Learning** | | | | | |
| **No.** | **Author** | **Outcome measures** | **Impact** | **Relevance to Geriatrics** | **Learning for the future** |
| 5 | Varkey et al. | Modified Maxwell-Sullivan questionnaire.  Aging Semantic Differential (ASD) | Statistically significant improvement in 6 of the 8 attitudes towards caring for elderly. Statistically significant increase in empathy towards elderly patients (23 out of 32 ASD questions). 93% of students would take the course if not mandatory. 98.8% of students stated the game added moderately - significantly to their knowledge and skills in older patient care. | First year medical students as introduction to geriatric curriculum and insight into problems experienced by the elderly. | Potential for the aging game to reinforce negative stereotypes, plan to provide more interaction and discussions about community-dwelling elderly and "successful aging". Only first years included. No longitudinal data. |
| 6 | Pacala et al. | Numeric student evaluations using five-point Likert scale.  Written comments | 93% rated the sessions overall as very good or excellent. However <50% of students’ evaluations available. Written comments supported 3 themes: mode of learning, attitudinal change, educational value and effect. The Aging game altered over the 10 years so evaluations were for different simulations although feedback remained positive. | Raise awareness of the field of Geriatrics. Enhance student understanding and contemplation of the aging experience and geriatric care. Inspire students to consider careers in Geriatrics | Longitudinal behavioural and attitudinal data beyond medical school would further explore any lasting effects of the Aging game |
| 7 | Lorraine et al. | Process evaluation of course (rate 1-9). Pre and posttest survey using the Aging Semantic Differential (ADS) Scale. Open-ended questions about the strengths and weaknesses of the exercise. | Average ratings of simulation exercise =7.36 within excellent range. The aging simulation exercise was the highest rated program in the geriatrics clerkship. Statistically significant improvement in attitudes towards the elderly on the ASD. Open-ended questions reported frustration, fatigue, anger, empathy for caregivers, paranoia, depression, embarrassment, fear, apprehension. | Part of a 2-week mandatory geriatric clerkship for 4th year students. | No longitudinal data. Helpful during other years of study. No formal assessment of empathy. |
| **Impact and Learning** | | | | | |
| **No.** | **Author** | **Outcome measures** | **Impact** | **Relevance to Geriatrics** | **Learning for the future** |
| 8 | Sari et al. | Student Information Form (demographics). BES measuring cognitive and emotional empathy. KAOPS. In-depth semi-structured Interviews to determine students' feelings, opinions and awareness regarding their experiences completing the aged simulation suit tasks. | Statistically significant increase in empathy and attitude scores in the high-score group only. 4 themes from interviews: awareness, anxiety, empathic approach and helplessness/desperateness. | Students were registered in a Geriatric Nursing Course. | Small sample. Students volunteered to participate, should require a compulsory geriatric nursing course that includes both theoretical and clinical practices throughout the entirety of the nursing education process.  Minimum scores of students were close to medium level, potential for future larger study including students with levels below medium level. |
| 9 | Demirtas & Basak | Pre-test all students:  Locally developed Descriptive Characteristics Form. KAOPS. Positive and Negative Ageism Scale (PNAS). Randomisation. 3-hour lecture. Control group post-lecture:  KOAP and PNAS Simulation group post-test:  30 minute Debrief session KOAP and PNAS Feedback analysis with face-to-face 15 minute interviews asking 5 semi-structured questions (n=49) | Intergroup comparison of KAOP and PNAS scores revealed a statistically significant difference between pretest and posttest scores in the simulation group.  Posttest KOAP and PNAS scores of the simulation and control groups were statistically significantly different. Feedback analysis determined 4 themes: Empathy Sorrow Awareness of geriatric nursing Entertainment | Used in the scope of Geriatric nursing. | Short time interval between pre and posttest scores. No longitudinal data. Author states only some ADLs included. Most participants were female. Recommended for inclusion in the nursing curriculum. Further studies to develop nursing students' positive attitudes toward older adults before the students have clinical practice and continuity of these studies should be maintained. Simulation could be an efficient strategy to attract the field of geriatric nursing to students and contribute to their careers. |
| **Impact and Learning** | | | | | |
| **No.** | **Author** | **Outcome measures** | **Impact** | **Relevance to Geriatrics** | **Learning for the future** |
| 10 | Koh et al. | Pre and Post-test interventions. 18-item UCLA-GKT. Singapore-modified 16-item UCLA-GAT. | Less positive attitudes at baseline among new curriculum students. New module improved both geriatric knowledge and attitudes. | Part of a 2-week Foundations in Geriatric Medicine module developed as part of a new Geriatric Medicine curriculum. | Single institution study. Validity of tools from Western settings being used in Asian settings with different education and healthcare systems. No longitudinal data, intention to re-assess groups in their final year.  Need for outcome studies on geriatric curriculum and assessment to identify ideal curriculum models in geriatrics. Develop and validate instruments to measure effectiveness of curricular innovations. |
| 11 | Tremayne et al. | Post-test evaluation tool. Five-point Likert scale. | 94% rated the session as good or excellent. 100% recommended the session for future students. Qualitative comments indicated that students were "treated differently", frustration, lack of insight from peers about the difficulties they were experiencing. | Session contributes to a study day which is part of the module "nursing approaches to enablement". | No longitudinal data. Only 2 suits in one size, need for more suits in varying sizes. The suit is based on a "common Japanese physique", although it can be adjusted some felt it was too small. Only nursing students, future interprofessional collaboration with other healthcare students. |
| 12 | McVey et al. | Postgame evaluation. Questionnaire. | 72% of students completed the evaluation. 75% of completed responses rated the geriatrics module as very good or excellent. Qualitative comments. | Simulation module as part of the required Introduction to Clinical Medicine course. | No longitudinal data. Concern of potentially reinforcing negative stereotypes, need to balance with presentations/post-exercise discussions around "successful aging". |
| **Impact and Learning** | | | | | |
| **No.** | **Author** | **Outcome measures** | **Impact** | **Relevance to Geriatrics** | **Learning for the future** |
| 13 | Robinson & Rosher | Pre and post simulation.  Aging Semantic Differential Scale (ASD). Post-class evaluation. Five-point Likert scale. | ASD scale indicated overall negative attitudes toward aging in this group. Highly significant change on the instrumental-ineffective domain/subscale of the ASD. Overall rating for class on Likert scale = 4.57 | Attempt to instill a sense of hope, possibility and potential for improvement. To appreciate how simple adaptions may allow an older adult to remain in an independent living arrangement. "Half-full" rather than "half-empty" approach toward older people | No longitudinal data. Students recommended the class be taught in 1st year so early education in geriatrics and prior to 1st clinical encounter with older patients. One-off session, no follow-up. Develop other courses targeting remaining attitudinal domains. Integrate older adults into course to gain social interaction. Future need for Interdisciplinary education. Coursework and clinical experiences focusing on measures that maintain and improve functioning should help students see challenges and possibilities for improvement in quality of life of elders. |
| 14 | Pacala et al. | Locally developed Characteristics questionnaire. Pre and posttest instrument assessed 4 domains (empathy, attitudes, knowledge, beliefs) with modifications from Maxwell & Sullivan's questionnaire & ASD.  Post-workshop student evaluation, qualitative open-ended items, quantitative 9-item 5-point Likert scale. | Aging Game participants significantly improved in empathy, attitudes and knowledge. Mean empathy score improved by 23%. Non-participants did not improve in any of the four domains. 91% of Aging Game participants rated the workshop as excellent or very good. Quantitative responses addressed empathy and attitudes toward caring for elderly patients. A few students highlighted frustration of dealing with the simulated progression of the game. | The exercise was offered as part of a workshop within a required six-week 4th year rotation in ambulatory medicine. The exercise occurred within the first 2 weeks of the rotation. Due to positive response the workshop was made a required component of the ambulatory medicine rotation. | Small sample size. Non-randomised design. Short-time interval for follow-up. Use of self-administered, unvalidated instruments. No longitudinal data. Author states the workshop should undergo further, more rigorous testing. |

Appendix 6: Different scales used to measure attitudes, knowledge and empathy

| **Scale** | **Study** | **Reference** |
| --- | --- | --- |
| Los Angeles Geriatric Attitudes Scale | Torkshavand et al. | 1. Torkshavand G, Khatiban M, Soltanian A. Simulation-based learning to enhance students’ knowledge and skills in educating older patients. Nurse Education in Practice 2020;42:102678. |
| Approaches to Dementia Questionnaire (ADQ) | De Abreu et al. | 1. de Abreu I, Hinojosa-Lindsey M, Asghar-Ali A. A Simulation Exercise to Raise Learners’ Awareness of the Physical and Cognitive Changes in Older Adults. Acad Psychiatry 2017;41:684–7. |
| Kiersma-Chen Empathy Scale (KCES),  Jefferson Scale of Empathy-Health Professions Students (JSE-HPS), Ageing Simulation Experience Survey (ASES) | Chen et al. | 1. Chen A, Kiersma M, Yehle K et al. Impact of the Geriatric Medication Game® on nursing students’ empathy and attitudes toward older adults. Nurse Education Today 2015;35:38–43. |
| Ageing Semantic Differential (ASD) | Varkey et al.  Lorraine et al.  Robinson et al.  Pacala et al. | 1. Varkey P, Chutka D, Lesnick T. The Aging Game: Improving Medical Students’ Attitudes Toward Caring for the Elderly. Journal of the American Medical Directors Association 2006;7:224–9. 2. Lorraine V, Allen S, Lockett A et al. (1998) “Sensitizing students to functional limitations in the elderly: an aging simulation.” *Family Medicine*, 30(1), pp. 15–18. 3. Robinson S, Rosher R. Effect of the “Half-Full Aging Simulation Experience” on Medical Students’ Attitudes. Gerontology &amp; Geriatrics Education 2001;21:3–12. 4. Pacala J, Boult C, Bland C et al. Aging Game Improves Medical Students’ Attitudes Toward Caring for Elders. Gerontology &amp; Geriatrics Education 1995;15:45–57. |
| Basic Empathy Scale (BES),  Kogan’s Attitude towards Older People Scale (KAOPS) | Sari et al. | 1. Sari D, Taskiran N, Baysal E et al. Effect of an aged simulation suit on nursing students’ attitudes and empathy. Eur Geriatr Med 2020;11:667–75. |
| Positive and Negative Ageism Scale (PNAS),  KAOPS | Demirtas & Basak | 1. Demirtas A, Basak T. Daily life activities simulation: Improving nursing students’ attitudes toward older patients. Jpn J Nurs Sci 2020;18, DOI: 10.1111/jjns.12375. |
| University of California Los Angeles (UCLA) Geriatric Knowledge Test (GKT),  A modified UCLA Geriatric Attitudes Test (GAT) | Koh et al. | 1. Koh G, Merchant R, Wee L et al. (2012) “The knowledge-attitude dissociation in geriatric education: Can it be overcome? ” *Annals of the Academy of Medicine, Singapore*, 41(9), pp. 383–389. Available at: https://doi.org/10.47102/annals-acadmedsg.v41n9p383. |
